# Quality Nutrition Care: Measuring Hospital Staff’s Knowledge, Attitudes, and Practices

**DOI:** 10.3390/healthcare4040079

**Published:** 2016-10-20

**Authors:** Celia Laur, Hannah Marcus, Sumantra Ray, Heather Keller

**Affiliations:** 1 School of Public Health and Health Systems, Faculty of Applied Health Sciences, University of Waterloo, Waterloo, ON N2L 3G1, Canada; cvlaur@uwaterloo.ca; 2 The Need for Nutrition Education/Innovation Programme, c/o MRC Elsie Widdowson Laboratory, University of Cambridge, Cambridge CB1 9NL, UK; sumantra.ray@mrc-ewl.cam.ac.uk; 3 Department of Clinical Nutrition, Grand River Hospital, Kitchener, ON N2G 1G3, Canada; hannah.marcus@grhosp.on.ca; 4 Schlegel-University of Waterloo Research Institute for Aging, Waterloo, ON N2J 0E2, Canada; 5 Department of Kinesiology, Faculty of Applied Health Sciences, University of Waterloo, Waterloo, ON N2L 3G1, Canada

**Keywords:** measurement, knowledge, attitudes and practices, hospital staff, nutrition care

## Abstract

Understanding the knowledge, attitudes, and practices (KAP) of hospital staff is needed to improve care activities that support the detection/prevention/treatment of malnutrition, yet quality measures are lacking. The purpose was to develop (study 1) and assess the administration and discriminative potential (study 2) of using such a KAP measure in acute care. In study 1, a 27-question KAP questionnaire was developed, face validated (n = 5), and tested for reliability (n = 35). Kappa and Intraclass Correlation (ICC) were determined. In study 2, the questionnaire was sent to staff at five diverse hospitals (n = 189). Administration challenges were noted and analyses completed to determine differences across sites, professions, and years of practice. Study 1 results demonstrate that the knowledge/attitude (KA) and the practice (P) subscales are reliable (KA: ICC = 0.69 95% CI 0.45–0.84, F = 5.54, *p* < 0.0001; P: ICC = 0.84 95% CI 0.68−0.92, F = 11.12, *p* < 0.0001). Completion rate of individual questions in study 2 was high and suggestions to improve administration were identified. The KAP mean score was 93.6/128 (range 51–124) with higher scores indicating more knowledge, better attitudes and positive practices. Profession and years of practice were associated with KAP scores. The KAP questionnaire is a valid and reliable measure that can be used in needs assessments to inform improvements to nutrition care in hospital.

## 1. Introduction

In 1974, Butterworth highlighted the essential role of quality nutrition care for health and recovery [1]. Since then, research has determined the prevalence of malnutrition and its impact on key health outcomes and issues [2,3,4,5,6], yet little research has attempted to improve its detection and treatment. As approximately 20%–50% of patients in acute care are malnourished [2,3] effective strategies to address this significant problem are needed. In 2013, the Alliance to Advance Patient Nutrition published a call to action for improving nutrition care in hospitals [7], which suggested that a comprehensive approach involving all staff was needed [2,8,9]. In response, a consensus based Integrated Nutrition Pathway for Acute Care (INPAC) was developed [10]. INPAC aims to address hospital malnutrition by incorporating evidence of best practice into a pathway specifying key care activities, such as nutrition screening at admission (e.g., with Canadian Nutrition Screening Tool (CNST)) [11] and diagnosing and triaging patients with the subjective global assessment (SGA) [12]. The INPAC provides guidance regarding how to implement best practices, considering both bottom-up (direct care staff) and top-down (policy and management level) approaches. INPAC emphases that all staff have a role to play in preventing, detecting, and treating malnutrition.

Implementing best practice requires a multifaceted approach, including education and training, as well as other behavior change techniques [13]. Before attempting to raise awareness on a particular topic through education, it is necessary to understand the environment, potentially using a knowledge, attitudes, and (self-reported) practices (KAP) questionnaire [14]. This type of questionnaire aims to measure what is “known, believed, and done in relation to a particular topic” [15] (p. 6). A KAP questionnaire can be used as part of a needs assessment before implementing best practice, such as improving nutrition care practices towards the ideal.

Staff-focused questionnaires used to date have been designed to detect gaps in nutrition knowledge [16,17] or attitudes and routines [18,19,20]. These assessments have demonstrated gaps between knowledge, attitudes, and practices, yet they have limitations. No questionnaire currently exists to adequately capture a broad target audience of healthcare professionals (hospital staff), or sufficiently address specific nutrition care activities focused on prevention, detection, and treatment of malnutrition.

A reliable questionnaire is required to understand the KAP of hospital staff in their provision of nutrition care. It is anticipated that such a questionnaire would demonstrate diversity among (1) sites; (2) professions; and (3) years of practice that could be used to inform behavior change strategies. A questionnaire such as this could be used as part of a needs assessment, identifying gaps in care and areas to focus the behavior change strategies, to ultimately impact patient health outcomes [16,17]. The aims of this manuscript are to: (1) describe the development of a KAP questionnaire for hospital staff regarding nutrition care (study 1); (2) to assess the administration and discriminative potential of this questionnaire (study 2). Preliminary results regarding differences between sites, professions, and years of practice are provided demonstrating the capacity of this questionnaire to discriminate between KA and P within these respondent characteristics.

## 2. Materials and Methods

### 2.1. Study 1: Development and Face Validation of KAP Questionnaire

An initial draft of the questionnaire was created to reflect key prevention, detection, and treatment activities consistent with INPAC, as well as incorporating nutrition knowledge and attitude domains from other applicable questionnaires and research [16,17,18,19,20,21,22]. A Likert scale was used for response options [23]. Knowledge and attitude (KA) questions had the same response categories and were treated in the same way conceptually and for scaling as it is difficult to distinguish between what is known and what is believed; categories included Strongly Disagree, Somewhat Disagree, Neutral, Somewhat Agree, and Strongly Agree. For the practice questions (P), a four-point scale was deemed appropriate and responses included Never, Sometimes, Often, Always, and Not Applicable. The draft questionnaire was reviewed independently by eight experts in the field. 

Cognitive interviews were then conducted with health professionals (n = 5; 2 dietitians, 1 diet technician, 1 food service manager, 1 nurse). Interview questions focused on the applicability, the wording (was it clear?), and the interpretation of the question (what did they think the question meant?). The questionnaire was deemed applicable for hospital staff with a clinical role, however was not applicable for food service workers, food service managers, or dietitians as too many of the questions were not relevant or, as with dietitians, their results would not be representative of the general staff on the unit.

### 2.2. Study 1: Test-Retest Reliability

Test-retest reliability demonstrates the stability of questions and that interpretation is consistent over time if no intervention occurs [23]. To address the issues of memory and maturation typically associated with test-retest reliability [23], a two-week period was chosen as the time between test administrations. Sample size calculations were based on a Pearson’s correlation coefficient (r) [23], which was used to estimate the intra-class correlation. With a sample of 60 staff members, a correlation among administrations of the questionnaire as small as r = 0.4 (two-sided test α = 0.05, β = 0.10 i.e., 90% power) could be determined [24,25].

Participants were recruited at a single hospital site using a display table in the cafeteria during a two-week period. An incentive ($5 gift card for a coffee shop) was provided for completing the questionnaire at two time points. Eligible participants were those with a clinical role and direct patient contact in any inpatient department of the hospital. Food service workers, food service managers, and dietitians were excluded as explained above. Each eligible participant consented to complete both questionnaires and responses were kept confidential. Two weeks after a participant had completed the initial hardcopy, the same questionnaire was sent by e-mail or mail for completion and return to the investigators. Up to four reminders were sent to participants over a six-week period to support completion.

### 2.3. Analysis

Kappa was calculated to determine reliability of individual questions and identify items requiring revision or removal [26]. KA questions (Strongly Disagree, Somewhat Disagree, and Neutral vs. Somewhat Agree and Strongly Agree) and P questions (Not Applicable and Never vs. Sometime, Often, and Always) were collapsed into two categories for analysis. Despite recruitment efforts, a lower number of respondents for both administrations resulted (test 1: n = 60, test 2 n = 35). Thus, a Kappa of 0.3 (fair) was used to determine reliability of individual questions [26] and potential items for removal prior to calculating subscale reliability. Level of agreement (total number of “matching” responses, i.e., those that provided the same answer in both questionnaires) was also determined and used in conjunction with the Kappa score to show reliability for individual questions. Kappa, level of agreement, and significance were considered together to determine if the individual question was reliable.

For determining total scale reliability, Intraclass Correlation Coefficient (ICC) was used. Two subscales were developed: the KA questions were separated from the P questions. Items (questions 1,8,13,15) that were negatively stated were reverse coded and the subscale total calculated so that a higher score indicated the more positive KA and P. For ICC, “fair to good agreement” is recognized as 0.61–0.8 and “excellent agreement” as 0.81–1.00 [24,25]. Analysis was completed using SPSS Version 23 (IBM SPSS Software, Chicago, IL, USA).

### 2.4. Study 2: Administration and Descriptive Analysis of KAP

The More-2-Eat (M2E) implementation project is a developmental evaluation designed to explore how INPAC activities can be implemented in five hospitals (one medical unit/hospital) in four provinces across Canada. An important component of INPAC implementation is to understand staff views and practices regarding nutrition care in order to provide direction on areas of focus and influence staff behavior change strategies. The M2E project provided the opportunity to further test how the KAP questionnaire for staff could be administered in the acute care setting and describe differences in KAP between profession and years in practice for KA and P. This testing provides information regarding how long it may take for a specified number of staff to complete the questionnaire, strategies for improving completion, and incentives required. Hospital staff do not feel they have time for questionnaires, however the information provided is important for identifying targets for behavior change. When deciding to use a KAP questionnaire as part of a needs assessment, it is important to understand the potential ways it can be used and how it can discriminate between specific groups of respondents.

The KAP questionnaire was completed at the five M2E sites to characterize the KAP of unit staff. The questionnaire was placed on Simple Survey (Outsidesoft Solutions Inc., Quebec, QC, Canada). Consent was provided by the hospital sites to send e-mail invitations to unit staff, facilitated by the M2E personnel seconded at the site. Reminders were sent regularly (e-mail and in person) until the quota (30/site) was complete (open from 30 September 2015 to 25 January 2016). All staff on the M2E unit were eligible to complete the questionnaire if they had a direct clinical role with patients, excluding dietitians.

Based on a 30-bed unit with approximately 30 nurses (full and part time) and 60 staff (estimate based on personal communication with sites), it was deemed feasible to obtain 30 responses per site for a total of n = 150 across the five sites. This was agreed as a conservative estimate based on the anticipated staffing levels, but also the expected challenges with recruitment, as identified in the administration in the test retest reliability study (study 1) at a single site. Thirty responses per site was also deemed adequate to understand the KAP for the unit staff to support strategies for education and training. This total is also consistent with Kaliyaperumal [16] who states the need to aim for a sample size of 200 with a reasonably high response rate.

### 2.5. Analysis

The mean KAP total, as well as the KA and P scores were calculated across all five sites. ANOVA was used to determine if there was a difference in scores among sites. Where no statistically significant differences were noted, samples were collapsed across sites to explore any associations between staff role (nurse vs. other) and years of practice as these were hypothesized to influence KA and P. It was also hypothesized that profession (nurse vs. other) and years of practice would influence KA and P scores. Discussion between researchers and M2E personnel from the five sites were held monthly to learn about survey recruitment challenges and strategies to overcome those challenges.

### 2.6. Ethics

Study 1 received ethics clearance through a University of Waterloo Research Ethics Board (UW REB) (ORE #: 20730). Approval for test-retest reliability was provided by the Tri-Hospital Research Ethics Board, through Grand River Hospital (THREB #2015-0571). Study 2 received clearance from a UW REB (ORE #: 20590) and by the ethics committees at each of the five hospitals as part of the ethics protocol for M2E.

## 3. Results

### 3.1. Study 1: Test Retest Reliability Results

Sixty participants were recruited and completed the first administration; 35 questionnaires were returned after the second administration. Demographic information is provided in Table 1. The Kappa, agreement, and significance were calculated (Table 2). The questions with Kappa below 0.3 and low agreement were noted, discussed, and minor edits were made prior to their use in Study 2. Even though some questions only had slight agreement, no questions were removed because they were all deemed necessary for understanding the KAP related to preventing, detecting, and treating malnutrition.

For subscale reliability, the KA had “fair to good reliability” (calculated ICC = 0.69 (95% CI 0.45–0.84), F = 5.540 (*p* < 0.001)) and P had “excellent reliability” (calculated ICC = 0.845 (0.68−0.92), F = 11.118 (*p* < 0.001)) [23,24]. It is noteworthy that, even considering the lower bound of the 95% confidence interval, both scales met our a priori criterion for a reliable measure.

Based on the adequate Kappa (0.3) for most of the individual questions, high agreement, and the relatively high ICC for KA and P subscales, the questionnaire was deemed reliable and appropriate for use.

### 3.2. Study 2: Administration Results

KAP questionnaires were completed at the five M2E sites and exceeded the original quota per hospital (n = 189). The survey remained open until all sites had reached 30 participants who completed the questionnaire and included their contact information. The time to complete 30 surveys ranged from 45–94 days (mean = 75 days). It should be noted that this period included Christmas (no recruitment), and that many sites reached the target before these dates. For M2E criteria, only respondents with contact information could contribute to the target of 30, thus these recruitment times may be inflated.

For recruitment of participants, in-person as well as e-mail reminders were used. It was found that some hospital staff did not have access to e-mail and requested hardcopies of the questionnaire. Some staff were also unaware that they had a hospital e-mail address. Due to issues of confidentiality within the units, use of hardcopies was not possible in this study. Access to a computer was also seen as a barrier.

There were very little missing data. Only four people did not answer five of the KA questions. For the practice questions, questions left blank were N/A (range from 12%–23%) and were treated as N/A rather than missing data. The highest proportion of responses was from Registered Nurses (35%) and Registered Practical Nurse/Licensed Practical Nurse (15%). As anticipated, Other Staff (25%) was also quite high. Demographic information of participants is presented in Table 3.

### 3.3. Study 2: Descriptive Results from More-2-Eat Sites

The mean KAP score from the five sites was 93.6/128 (Range 51–124). For Site A, the mean score was 92/128 (Range 63–114); for Site B 93.7/128 (Range 55–120); for Site C 91.9/128 (Range 56–124); for Site D 94.7/128 (Range 66–116); and for Site E 94.1/128 (Range 51–114). There was no significant difference among sites for the total KAP score (F (4,184) = 0.379, *p* = 0.823). Sites were collapsed to determine if differences existed among professional groups and years of practice.

Breakdown of proportion of participants in each response category per question are included in Table 4 for the KA questions and Table 5 for P questions. Most (88%; n = 166) respondents thought that nutrition was important, however only 62% always knew when to refer to a dietitian (n = 118), but 80% (n = 152) knew how to refer. A little more than half (58%; n = 110) reported knowing when a patient was at risk of malnutrition or was malnourished and a similar proportion (55%; n = 104) reported often/always helping a patient open food packages, and providing eating assistance when needed (49%; n = 92). However, only 35% of respondents reported realigning their tasks so as not to interrupt a patient during their meal time.

When comparing nurses (n = 89) to other hospital staff (n = 111), there was a significant difference in total KAP score (nurses = 99.5/128; other = 88.3/128); t (187) = 5.89, *p* = 0.000), the KA score (nurses = 80.1/100; other = 76.4/100; t (187) = 2.677, *p* = 0.008), and the P score (nurses = 19.4/28; other = 11.9/28; t (187) = 7.71, *p* = 0.000). This indicates that nurses had more/better knowledge and attitudes and were more likely to report care behaviors that supported the detection, prevention, and treatment of malnutrition than non-nursing direct care staff.

There was no significant difference for total KAP score for years in practice. A significant difference was found for years in practice to KA score (F (5182) = 2.87, *p* = 0.016) with those practicing for 21–30 years having the highest mean KA score (81.85 (CI 79.03–84.67)) and those in the 2–5 years practicing category having the lowest mean KA score (74.02 (CI 70.89–77.16)). A significant difference was also found for years practicing and mean P score (F (5182) = 3.276, *p* = 0.007) with those practicing less than 2 years having the highest mean P score (18.00 (CI 14.39–21.61)) and those practicing for more than 31 years having the lowest (10.31 (CI 6.10–16.51)).

## 4. Discussion

In Study 1, a valid and reliable questionnaire was developed to assess nutrition KAP applicable for a wide variety of healthcare professionals who work in the hospital setting. The intent was to have a questionnaire that reflected quality nutrition care practices, and could be used as one of several instruments for a needs assessment when using behavior change to implement nutrition care improvements. The questionnaire needed to be applicable to hospital staff who do not necessarily see themselves as having a direct role in nutrition care, yet are still involved in nutrition care, such as opening food packages, making food available on the unit for patients, and avoiding mealtime interruptions. Scaling results indicate that although improvements can be made and the sample size was small, the questionnaire was sufficiently reliable for use. The questionnaire is designed for use by hospitals to provide direction and feedback regarding which areas of their own staff behavior to focus on when optimizing nutrition care. It is recommended that future users of this KAP questionnaire consider which questions are applicable to their needs and context. The Kappa values for individual questions provide some assurance of item vs. scale reliability.

Study 2 provided information regarding how best to administer the questionnaire in acute care settings, while retaining anonymity of respondents. Although barriers to completion were highlighted, several strategies were used to increase completion. Potential strategies included having hardcopies available on the unit (keeping in line with confidentially agreements), or only sending the questionnaire when no other hospital wide survey was underway. Incentives (i.e., entry into a draw, snacks, verbal encouragement), verbal reminders, and competition between units were all strategies used to increase completion rates. No complaints or concerns with respect to length of the questionnaire were reported.

Results from study 2 provide a sense of the capacity of the KAP questionnaire to discriminate between KA and P among professional groups and across years of practice, which lends further credibility to this measure. Prevalence of key items also confirms a need for further education and training to improve nutrition care in hospital; although a high percentage (88%) of staff already believe nutrition was important. Unfortunately, this belief did not always translate into practice as only 28% always checked to see that a patient had everything they needed to eat, and only 43% always helped to open food packages. Although the KA scores were relatively high for this group, the P scores demonstrate room for improvement. For example, proponents of “protected mealtimes” suggest decreasing mealtime interruptions [27,28,29,30], yet only 35% of M2E hospital staff arrange their tasks to minimize this interruption. Food intake is an important factor for determining length of stay, and 82% agreed/strongly agreed that monitoring food intake was important, yet this was not always done in practice. 

Several studies have shown that education can increase knowledge, yet this does not mean that it will improve practice immediately, as changing behavior is part of a continuous process [31,32]. For this reason, it is important to use a multi-faceted approach to behavior change that provides education and/or training, while also working on other components, such as having an environment conducive to the change [15]. If the processes are not in place for staff to apply their knowledge, education that increases knowledge is unlikely to influence practice.

Exploratory analyses comparing groups of staff based on their discipline and years of practice suggest potential differences in KAP worthy of further investigation. For years in practice, it was not surprising to have more experience relating to higher KA, however it was unexpected to have this equating to lower P scores. Since most differences were expected, it reinforced the need to focus on education of staff as well as ensuring the processes are in place to practice what is learned. Conclusions with respect to the identified associations in this analysis cannot be made until more diverse samples with greater generalizability are assessed with the KAP questionnaire. However, locally sensitive data can be used for bespoke local solutions, which can subsequently add to the body of regionally effective best practices since there is no “one size fits all” solution in health systems improvement.

### 4.1. Limitations

Although identified to be reliable, the KAP questionnaire could benefit from further development. Due to the time restrictions of the M2E project, pretesting of the questionnaire was limited. Future analysis should include cognitive interviews with physicians and allied health to ensure that questions are fully understood. After completion of the M2E project, further items to support improved nutrition care practices may become evident for consideration and inclusion in the next version of the questionnaire. Test-retest reliability should be conducted on any revised version of the questionnaire.

Analysis of the M2E results examined differences across professions (nurses versus other professions), differences based on years in practice, and differences between sites; however, the sample size is not designed for these individual comparisons and any statistically significant differences should be interpreted with caution. A larger sample size was deemed unrealistic based on limits of the M2E study, as well as learnings from study 1.

It is important to note that these are self-perceived practices and may not be representative of what occurs in real life. There are also many more questions that could be asked, but given the busy schedule of hospital staff, the questionnaire had to be completed within a maximum of 5–10 min. Given these limitations, the questionnaire was still deemed sufficient to use within M2E to determine the KAP environment of each site.

### 4.2. Using the Results

This questionnaire provides important information to inform gaps in KAP and areas to focus behavior change strategies for improving staff nutrition care. Within M2E, sites received their results and the overall average scores from across the five sites. This technique could be used by any hospital to compare between units. This questionnaire can be used as an evaluation instrument, as it can be re-administered after behavior change efforts have been made to see if there is a change over time. In M2E, the questionnaire will be used again at the end of the project as a way to examine if there is any change in KAP after one year of INPAC implementation. If the same participants complete the questionnaire, intra-individual changes over time can be assessed. 

## 5. Conclusions

The KAP questionnaire is a face valid and reliable questionnaire that has the potential to support understanding of staff KA and P with respect to nutrition care. The questionnaire can be used as a needs assessment in an educational project to improve these aspects. However, it may need to be adapted based on the context and applicability of questions within the needs assessment. Strategies for recruitment within acute care are likely to be applicable across several contexts. Results from M2E sites indicated that KA scores are higher than P scores, suggesting that education is not sufficient to change staff behavior with respect to best practice for nutrition care in hospital. Use of KAP questionnaires may also improve awareness in respondents as well as hospital management who approve its use. Overall, this questionnaire provides direction and feedback, which can be used by hospitals and researchers aiming to optimize nutrition care in hospital.

## Figures and Tables

**Table 1:** Study 1: Demographics for test retest reliability participants (n = 35).

Demographics	N (Percent)
Profession	
Registered Nurse	11 (31%)
Registered Practical Nurse/ Licensed Practical Nurse	2 (6%)
Attending Physician	1 (3%)
Physiotherapist/Occupational Therapist	4 (11%)
Resident	1 (3%)
Other	16 (46%)
Employment	
Full Time	23 (66%)
Part Time	11 (31%)
Casual	1 (3%)
Years Employed	
Less than 2 years	6 (17%)
2–5 years	6 (17%)
6–10 years	7 (20%)
11–20 years	8 (23%)
21–30 years	6 (17%)
31+ years	2 (6%)
Age	
<30 years	10 (29%)
30–39 years	9 (26%)
40–49 years	10 (29%)
50–59 years	5 (14%)
60+ years	1 (3%)
Gender	
Female	33 (94%)
Male	2 (6%)

Note: this table only includes results for participants who completed both administrations of the questionnaire.

**Table 2:** Study 1: Test retest reliability of the KAP questionnaire for individual questions.

Question	n	Kappa	Agreement	Sig.
Please rate your agreement with the following statements Strongly Disagree; Somewhat Disagree; Neutral; Somewhat Agree; Strongly Agree				
1. Nutrition is not important to every patient’s recovery in hospital ^+^	34	0.313	26/34	0.033
2. All patients should be screened for malnutrition at admission to hospital	33	0.713	30/35	0.000
3. A patient’s weight should be taken at admission	34	0.269	30/34	0.117
4. All staff involved in patient care can help set up the tray, open packages, etc.	34	0.197	23/34	0.248
5. All staff involved in patient care can provide hands-on assistance to eat when necessary	34	0.401	24/34	0.016
6. Malnutrition is a high priority at this hospital	33	0.471	24/33	0.003
7. Giving malnourished patients an adequate amount of food will enhance their recovery	33	0.436	29/33	0.009
8. All malnourished patients require individualized treatment by a dietitian ^+^	34	0.301	28/34	0.071
9. I have an important role in promoting a patient’s food intake	32	0.463	23/32	0.004
10. Monitoring food intake is a good way to determine a patient’s nutritional status	34	0.217	24/34	0.152
11. Interruptions during the meal can negatively affect patient food intake	35	0.643	31/35	0.000
12. Promoting food intake to a patient is every staff member’s job	35	0.340	25/35	0.043
13. Nutritional care of a patient is only the role of the dietitian ^+^	35	0.525	32/35	0.002
14. Malnourished patients who are discharged need follow up in the community	35	0.525	32/35	0.002
15. A patient’s weight is not necessary at discharge ^+^	34	0.209	26/34	0.184
Please rate your agreement with the following statements Strongly Disagree; Somewhat Disagree; Neutral; Somewhat Agree; Strongly Agree				
1. I always know when to refer to a dietitian	33	0.436	24/33	0.012
2. I know how to refer to a dietitian	34	0.672	29/34	0.000
3. I know when a patient is at risk of malnutrition or is malnourished	34	0.712	29/34	0.000
4. I know some strategies to support food intake at meals	34	0.580	27/34	0.001
5. I need more training to better support the nutrition needs of my patients	34	0.395	24/34	0.020
Please rate how often you DO the following Never; Sometimes; Often; Never; N/A				
1. Check the patient has all that they need to eat (e.g., dentures, glasses)	33	0.816	30/33	0.000
2. Help a patient with opening food packages	33	0.807	30/33	0.000
3. Assist a patient to eat if they need help	33	0.637	27/33	0.000
4. If permitted, encourage a patient’s family to bring food from home for the patient	32	0.808	29/32	0.000
5. Visit and check a patient during their meal time to see how well they are eating	33	0.573	26/33	0.001
6. Realign my tasks so I do not interrupt a patient during their meal time	33	0.518	25/33	0.002
7. At discharge of a malnourished patient, provide the patient or family with nutrition education material	32	0.167	22/32	0.346

Note: The number of questionnaires returned is out of a possible n = 60, yet not everyone completed all questions which accounts for the discrepancy across the n values. Kappa (0.3 considered “fair”) shows reliability of the individual question. Agreement demonstrates the number of people that provided the same answer in both questionnaires. ^+^: Reverse Coded; Sig.: Significance.

**Table 3:** Study 2: Demographic information of the hospital staff across five sites.

Profession (n = 189)	Percentage of Staff (n)
Registered Nurse	31% (58)
Registered Practical Nurse/Licensed Practical Nurse	15% (28)
Dietetic Technician	0.5% (1)
Health Care Aide/Personal Support Worker	5% (9)
Physiotherapist/Occupational Therapist	9% (17)
Speech-Language Pathologist	4% (8)
Attending Physician	6% (11)
Other	25% (48)
**Employment (n = 188)**	
Full Time	63% (119)
Part Time	29% (55)
Casual	7% (14)
**Years Employed (n = 187)**	
Less than 2 years	10% (19)
2–5 years	24% (45)
6–10 years	21% (40)
11–20 years	19% (36)
21–30 years	18% (34)
31+ years	7% (13)
**Age (n = 189)**	
less than 30 years of age	23% (43)
30–39 years of age	26% (48)
40–49 years of age	26% (48)
50–59 years of age	21% (40)
60 years of age	5% (9)
**Gender (n = 189)**	
Female	86% (162)
Male	14% (27)

**Table 4:** Study 2: Proportion of responses for knowledge/attitude questions (N = 189).

Questions	Strongly Disagree	Somewhat Disagree	Neutral	Somewhat Agree	Strongly Agree	Missing	Mean (out of 5)	Median (out of 5)
Please rate your agreement with the following statements:								
1. Nutrition is not important to every patient’s recovery in hospital *	12 (6%)	2 (1%)	0 (0%)	9 (5%)	166 (88%)	0	4.7	5
2. All patients should be screened for malnutrition at admission to hospital	6 (3%)	6 (3%)	21 (11%)	63 (22%)	93 (49%)	0	4.2	4
3. A patient’s weight should be taken at admission	7 (4%)	5 (3%)	10 (5%)	36 (19%)	131 (69%)	0	4.5	5
4. All staff involved in patient care can help set up the tray, open packages, etc.	7 (4%)	11 (6%)	14 (7%)	30 (16%)	127 (67%)	0	4.4	5
5. All staff involved in patient care can provide hands-on assistance to eat when necessary	8 (4.2%)	20 (11%)	20 (11%)	52 (28%)	89 (47%)	0	4.0	4
6. Malnutrition is a high priority at this hospital	9 (5%)	25 (13%)	48 (25%)	69 (37%)	38 (20%)	0	3.6	4
7. Giving malnourished patients an adequate amount of food will enhance their recovery	5 (3%)	8 (4%)	16 (9%)	59 (31%)	101 (53%)	0	4.3	5
8. All malnourished patients require individualized treatment by a dietitian *	108 (57%)	58 (31%)	12 (6%)	7 (4%)	4 (2%)	0	1.6	1
9. I have an important role in promoting a patient’s food intake	8 (4%)	13 (7%)	33 (17.5%)	61 (32%)	74 (39%)	0	4.0	4
10. Monitoring food intake is a good way to determine a patient’s nutritional status	3 (2%)	13 (7%)	18 (10%)	80 (42%)	75 (40%)	0	4.1	4
11. Interruptions during the meal can negatively affect patient food intake	2 (1%)	6 (3%)	14 (7%)	80 (42%)	87 (46%)	0	4.3	4
12. Promoting food intake to a patient is every staff member’s job	7 (4%)	8 (4%)	24 (13%)	59 (31%)	91 (48%)	0	4.2	4
13. Nutritional care of a patient is only the role of the dietitian *	11 (6%)	12 (6%)	18 (10%)	57 (30%)	91 (48%)	0	4.1	4
14. Malnourished patients who are discharged need follow up in the community	3 (2%)	7 (4%)	10 (5%)	70 (37%)	99 (52%)	0	4.4	5
15. A patient’s weight is not necessary at discharge *	5 (3%)	17 (9%)	54 (29%)	59 (31%)	54 (28%)	0	3.7	4
16. I always know when to refer to a dietitian	8 (4%)	32 (17%)	27 (14%)	87 (46%)	31 (16%)	4 (2%)	3.5	4
17. I know how to refer to a dietitian	8 (4%)	14 (7%)	11 (6%)	48 (25%)	104 (55%)	4 (2%)	4.1	5
18. I know when a patient is at risk of malnutrition or is malnourished	6 (3%)	36 (19%)	33 (18%)	85 (45%)	25 (13%)	4 (2%)	3.4	4
19. I know some strategies to support food intake at meals	5 (3%)	25 (13%)	36 (19%)	90 (48%)	29 (15%)	4 (2%)	3.5	4
20. I need more training to better support the nutrition needs of my patients	9 (5%)	17 (9%)	30 (16%)	77 (41%)	52 (28%)	4 (2%)	3.7	4
Total score (out of 100)	N/A	N/A	N/A	N/A	N/A	N/A	78.2	80

*: These are negative questions and the scoring was reversed: Strongly Disagree (5); Somewhat Disagree (4); Neutral (3); Somewhat Agree (2); Strongly Agree (1); Blank (0). A higher score indicates more positive knowledge/attitude. For example, in the first question 1, 4.7/5 means that more people think that nutrition *is* important. For question 8, 1.6/5 means that more people believe that all malnourished patients require individualized treatment by a dietitian.

**Table 5:** Study 2: Proportion of responses for practice questions (N = 189).

Questions	Never	Sometimes	Often	Always	N/A or Blank	Mean (out of 4)	Median (out of 4)
Please rate how often you DO the following:							
1. Check the patient has all that they need to eat (e.g., dentures, glasses)	22 (12%)	32 (17%)	47 (25%)	53 (28%)	35 (18.5%)	2.3	3
2. Help a patient with opening food packages	7 (4%)	35 (19%)	43 (23%)	81 (43%)	23 (12%)	2.8	3
3. Assist a patient to eat if they need help	33 (18%)	30 (6%)	34 (18%)	60 (32%)	32 (17%)	2.3	2
4. If permitted, encourage a patient’s family to bring food from home for the patient	17 (9%)	48 (25%)	55 (29%)	42 (22%)	27 (14%)	2.4	3
5. Visit and check a patient during their meal time to see how well they are eating	34 (18%)	33 (18%)	39 (21%)	45 24%)	38 (20%)	2.1	2
6. Realign my tasks so I do not interrupt a patient during their meal time	22 (12%)	59 (31%)	43 (23%)	37 (20%)	28 (15%)	2.2	2
7. At discharge of a malnourished patient, provide the patient or family with nutrition education material	83 (44%)	36 (19%)	14 (7%)	13 (7%)	43 (23%)	1.3	1
Total score (out of 28)	N/A	N/A	N/A	N/A	N/A	15.4	17
